# Delivery and uptake of free and liposome-encapsulated cholesterol-conjugated antisense oligonucleotides in Atlantic salmon sperm: insights from high-resolution imaging

**DOI:** 10.1186/s12917-026-05465-0

**Published:** 2026-04-17

**Authors:** Jaya Kumari Swain, Deanna Lynn Wolfson, Trilochan Swain, Gøril Eide Flaten, Nataša Škalko-Basnet, Balpreet Singh Ahluwalia, Helge Tveiten

**Affiliations:** 1 Nofima AS, Muninbakken 9-13, Tromsø, 9291 Norway; 2 Drug Transport and Delivery Research Group, Department of Pharmacy, UiT The Arctic University of Norway, Universitetveien 57, Tromsø, 9037 Norway; 3 Present Address: Department of Physics and Technology, UiT The Arctic University of Norway, Tromsø, 9037 Norway; 4 Present Address: Norwegian College of Fishery Science, Faculty of Biosciences, Fisheries and Economics, UiT The Arctic University of Norway, Tromsø, 9037 Norway

**Keywords:** Sperm, Liposomes, Delivery, Morpholino, Atlantic salmon

## Abstract

The development of efficient methods for large-scale delivery of antisense molecules to Atlantic salmon (*Salmo salar* L.) eggs remains a significant challenge, limiting the application of antisense technologies in salmon aquaculture at an industrial scale. This study aimed to establish a proof of concept for a non-invasive, accessible method for delivering antisense oligonucleotides (ASOs) to salmon eggs using sperm cells as a delivery vehicle. Salmon spermatozoa were incubated for 24 h at 4 °C with a fluorescently labeled and cholesterol-conjugated antisense morpholino oligonucleotide (MO) in two formulations: cholesterol-conjugated antisense morpholino free in solution (F-MO) and cholesterol-conjugated antisense morpholino encapsulated into liposomes (L-MO). The binding and internalization patterns of F-MO and L-MO in spermatozoa were evaluated using fluorescence super-resolution microscopy. Both F-MO and L-MO incubations resulted in fluorescent binding in nearly 100% of the sperm cells, with distinct preferences for the head and tail regions of the sperm membrane. Notably, L-MO demonstrated superior internalization into the sperm head, as evidenced by higher fluorescence intensity within the head region compared to F-MO. Fertilization trials confirmed the successful transfer of L-MO to the egg, as indicated by visible fluorescence in the zygote. Moreover, incubation with either MO formulation did not adversely affect fertilization success or embryonic development up to the “eyed stage” under the experimental set-up conditions. In summary, high-resolution microscopy revealed enhanced uptake and internalization of L-MO compared to F-MO, enabling delivery and visualization of fluorescence in the one-cell embryo post-fertilization. These findings highlight the potential of sperm cells as a viable delivery vehicle for non-invasive transfer of MO oligonucleotides to salmon eggs. However, further research is required to optimize this approach and evaluate its scalability. Thus, this study provides a foundation for the development of cost-effective and practical ASO-based technologies for application in salmon aquaculture.

**Supplementary Information:**

The online version contains supplementary material available at 10.1186/s12917-026-05465-0.

## Introduction

The application of antisense technologies has emerged as a powerful tool for gene modulation in various biological systems, including aquaculture species. Antisense oligonucleotides (ASOs), such as morpholino oligonucleotides (MOs), are synthetic molecules designed to specifically bind to target RNA sequences, thereby modulating targeted gene expression [1, 2]. Morpholino oligonucleotides has been tested in ovulated eggs of several fish species including zebrafish, coho salmon and sablefish as an alternative technology for gene silencing and sterilization [3]. Despite their potential, the large-scale delivery of ASOs to Atlantic salmon (*Salmo salar* L.) eggs remains a significant challenge, limiting their broader application in salmon aquaculture for gene manipulation. The development of efficient, non-invasive, and scalable delivery methods that is not modifying the organism’s DNA, is essential to harness the full potential of antisense technologies in salmon aquaculture and aquaculture in general [3].

Traditional methods for delivering ASOs to fish embryos often involve microinjection. Although effective, microinjection is labor-intensive, technically demanding, and unsuitable for large-scale applications in aquaculture. Therefore, alternative delivery strategies that are both efficient and practical are urgently needed. One promising approach is the use of sperm cells as delivery vehicles for ASOs. Sperm-mediated delivery offers several advantages, including its non-invasive nature, cost-effectiveness, scalability, and the potential for direct and natural transfer of targeted molecules to the egg during fertilization [4]. Spermatozoa have been extensively studied as natural transporters for gene or exogenous DNA transfer in transgenic animal technologies, with several approaches developed [5]. However, most of this research has focused on mammals, with limited studies in fish species [5–8]. Furthermore, the efficiency and mechanisms of sperm-mediated molecular transfer remain subjects of ongoing investigation and debate [9–14].

Liposome encapsulation has been widely explored as a strategy to enhance the delivery efficiency of nucleic acids and other biomolecules [15, 16]. Liposomes can protect ASOs from degradation, improve cellular uptake, and facilitate targeted delivery. In recent years, there has been growing interest in the use of nanomaterials for reproductive biology, particularly in delivering various molecular cargo into gametes to improve the efficacy of research techniques and expand their applications [17, 18]. In mammals, liposomes have been used for drug delivery and gene transfer due to their low toxicity and ability to facilitate the internalization of molecular cargo [19–22]. Liposome-mediated gene transfer via sperm has been successfully demonstrated in several mammalian species, including rabbits, chickens and cattle [4]. However, the application of liposome-mediated delivery in aquatic species, particularly teleost fish, remains largely unexplored.

Previously, we demonstrated the feasibility of liposome mediated molecular delivery to ovulated egg through co-incubation [23]. Building on this experience, it was hypothesized that sperm cells could serve as targeted delivery vehicles for ASOs, delivering their molecular cargo directly to the zygote, post fertilization. In this study, we aimed to establish a proof of concept for using salmon sperm as a vehicle for ASO delivery to the ovulated Atlantic salmon eggs, bypassing the need for direct treatment or co-incubation with eggs. Specifically, we investigated the uptake and delivery efficiency of two forms of fluorescently labeled standard control morpholino oligonucleotides conjugated to a cholesterol moiety (MO): “Naked/unencapsulated” MO, free in solution (F-MO) and liposome-encapsulated MO (L-MO). High-resolution fluorescence microscopy was employed to evaluate cell binding, localization, and internalization patterns of F-MO and L-MO in salmon spermatozoa. Fertilization trials were conducted to assess spermatozoa functionality and the ability of MO-loaded sperm to deliver molecular cargo into eggs during fertilization. The developmental potential of embryos fertilized with MO-loaded sperm was also monitored to ensure that the treatment did not adversely affect embryogenesis.

To the best of our knowledge, this is the first study to investigate the uptake and distribution of unencapsulated and liposome-encapsulated cholesterol-conjugated fluorescent oligonucleotides in salmon sperm using high-resolution imaging. Our findings provide valuable insights into the potential of sperm-mediated molecular delivery as a non-invasive and scalable approach for antisense technology in aquaculture.

## Materials and methods

### Morpholino Oligos (MO) and experimental setup

**Fig. 1:**
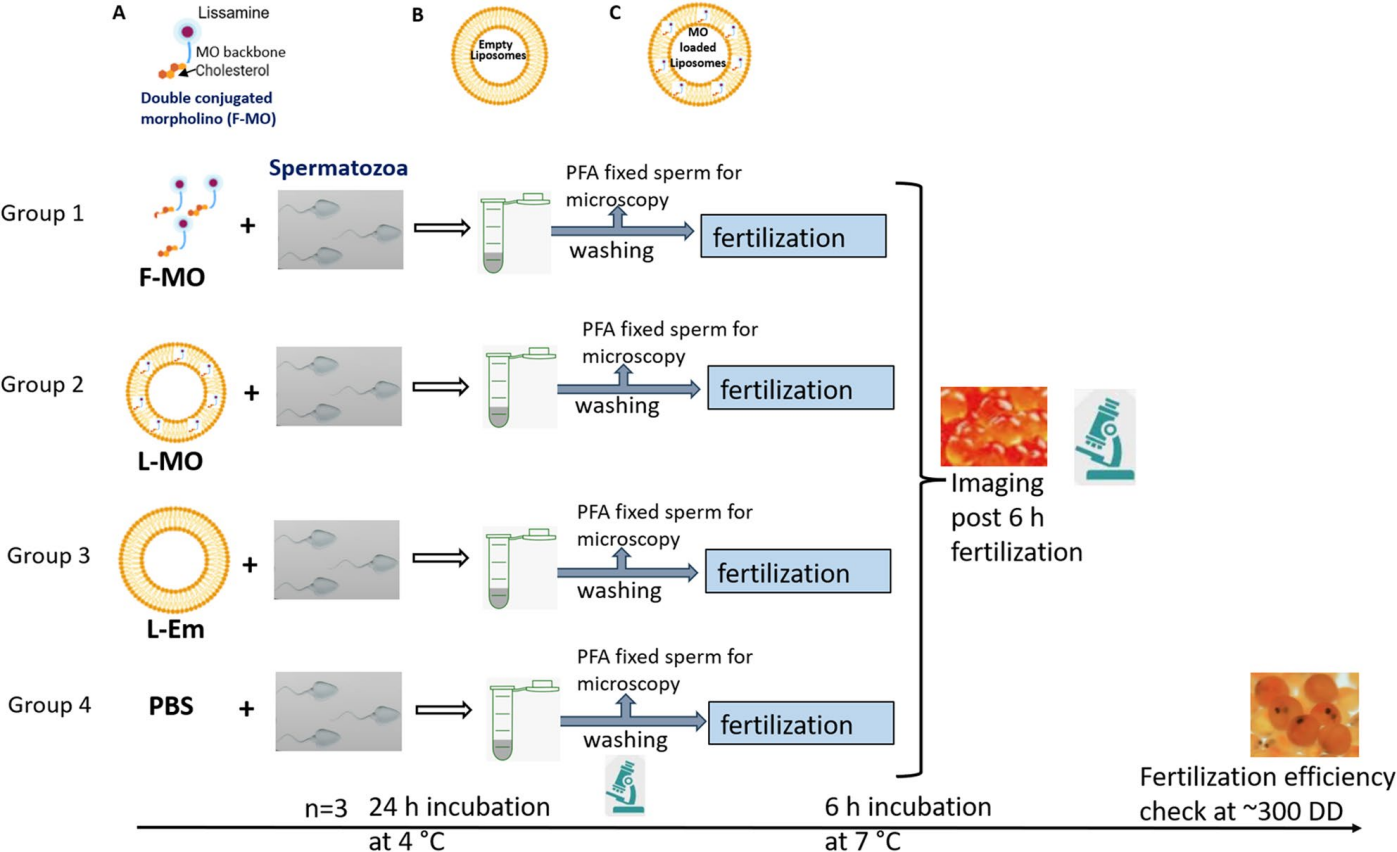
Schematic representation of the experimental setup. **A** showing schematic representation of double modified “standard control” morpholino oligonucleotide (MO) conjugated with 3’cholesterol and the fluorescent dye lissamine. **B** showing unilamaller empty liposomes. **C** showing liposomes loaded with double modified MO

A double modified “standard control” morpholino oligonucleotide (MO), which does not target any specific gene, was conjugated with 3’cholesterol and the fluorescent dye lissamine (MW: 9923 Da) and custom synthesized by Gene Tools Ltd. (Summerton Way, Philomath, USA). The non-specific control antisense sequence used in this study was ‘CCTCTTACCTCAGTTACAATTTATA’. The cholesterol moiety was incorporated to enhance cellular uptake efficiency of the fluorescent MO and to increase the lipophilic nature, facilitating efficient encapsulation within liposome [24, 25]. In this experiment, we intended to deliver lissamine labelled and cholesterol conjugated antisense MO through two different approaches ‒ cholesterol and lissamine conjugated MOs”free” in the incubation solution (F-MO) and F-MOs encapsulated into liposomes (L-MO). The detailed experimental plan is shown in Fig. 1.

### Preparation of liposome-encapsulated MO (L-MO)

Liposomes without (L-Em) or with encapsulated cholesterol-lissamine labelled MO (L-MO) were prepared using the film hydration method [26]. Conventional liposomes were composed of phosphatidylcholine (PC) from soybean lecithin (Lipoid S 100, > 94% PC) and cholesterol in a 9:1 (w/w) ratio, with a total lipid content of 495 mg. The lipids and fluorescently labeled MO (1 mol %) were dissolved in 25 mL of methanol. The organic solvent was then evaporated under reduced pressure using a rotary evaporator (Büchi Rotavapor R-124, Büchi Labortechnik, Switzerland) at 45 °C and 50 mm Hg for 3 h to form a thin lipid film. The resulting lipid film was hydrated with 10 mL of phosphate-buffered saline (PBS, pH 7.4) to produce liposomal dispersions. The liposomal suspensions were stored at 4–8 °C overnight before further use.

### Characterization of liposomes

Liposomes (L-MO) (10 mL; 49.5 mg/mL) were transferred to 20 mL beaker and placed in an ice bath. Using an ultrasonic processor (500 W, Sigma–Aldrich, St. Louis, MO, USA) set to 40% amplitude, the liposomes were exposed to ultrasonic irradiation for 5 cycles of 2 min each to obtain smaller unilamellar liposomes (~ 75–100 nm in diameter). The sonicated liposomes were stored at 4–8 °C for at least 6 h before further use. Empty liposomes (L-Em) were subjected to similar processing. Naked or unencapsulated MO (MW: 9923 Da) was separated from liposome-encapsulated MO (L-MO) using Nanosep 30 K ultrafiltration devices (MWCO 30 kDa, Pall Life Sciences, NY, USA). The liposomal suspension was washed twice with PBS to remove unencapsulated MO, and the purified L-MO was used for further characterization. The particle size distribution of the liposomes was measured by photon correlation spectroscopy (PCS) using a Submicron Particle Sizer (Model 370, Nicomp, Santa Barbara, CA, USA). Zeta potential measurements were performed using a Malvern Zetasizer Nano Z (Malvern, Oxford, UK) as described by Jøraholmen et al. [27]. The encapsulation efficiency (EE) of the fluorescent MO was calculated using the formula:

$$\%\mathrm{EE}=\left[\left(\mathrm{Total}\;\mathrm{MO}\;\mathrm{added}-\mathrm{unencapsulated}\;\mathrm{MO}\right)/\mathrm{Total}\;\mathrm{MO}\;\mathrm{added}\right]\times100$$

The L-MO were stored at 4 °C and used in experiments within 7 days of preparation.

### Co-incubation with salmon sperm

Milt from a single male Atlantic salmon (approx. 14 kg) were obtained from AquaGen AS, Trondheim, Norway and were shipped overnight at 4 °C in a sealed plastic Ziploc^®^ bags. For the co-incubation experiment, L-MO and F-MO were added to yield a final 50 µM concentration of fluorescent conjugated MOs to each tube containing 300 µl of sperm (2.6 × 10^8^ cells). The mixed solution was incubated at 4 °C for 24 h with gentle shaking. The experiment was conducted in triplicates. For the control group, 300 µL of sperm samples were treated under identical conditions, except that PBS and L-Em were used in place of F-MO/L-MO. After incubation, the spermatozoa were washed twice with PBS by centrifugation at 300 x g for 4 min at 4 °C. The washed sperm was then reconstituted to the original milt volume of ~ 350 µL and checked for viability before fertilization. For fertilization, 100 ul of sperm per replicate (~ 7.4 × 10^7^ cells) in 25 mL of water was used with eggs from a single female (also AquaGen AS, Trondheim, Norway) according to Kumari et al. (2017) [23] (see below). The remaining 50 µL of sperm were fixed in 4% paraformaldehyde (PFA) and washed three times with PBS before being used for high resolution fluorescence microscopy studies.

### Microscopy preparation and acquisition

**Fig. 2:**
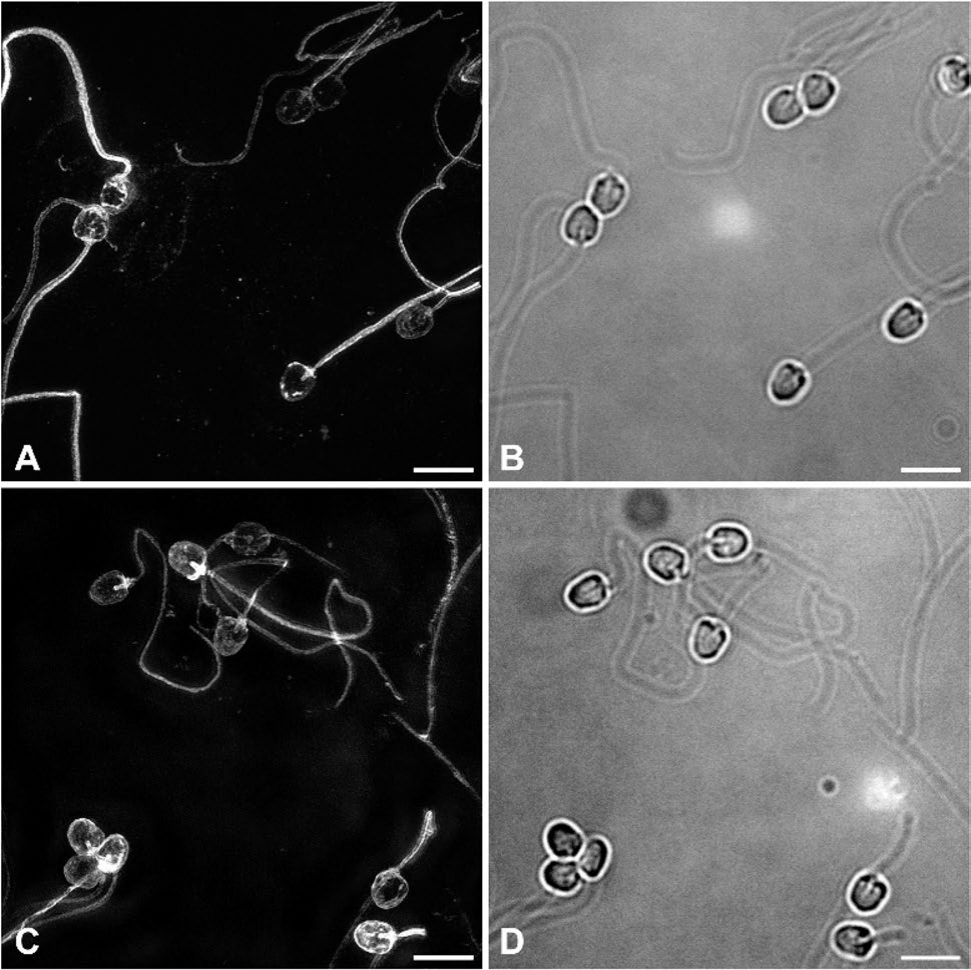
Representative images showing almost all salmon sperm are labeled using L-MO and F-MO. Salmon sperm treated with liposomal, L-MO (**A**, **B**) or free morpholino, F-MO (**C**, **D**) were imaged using fluorescence super-resolution microscopy (**A**, **C**) and brightfield microscopy (**B**, **D**). The brightfield images confirm that nearly all sperm treated with the cholesterol-conjugated fluorescent MO, in either free or liposomal form, is labeled and visible in the fluorescent images. Control sperm as shown in Supplementary Fig. 2 had fluorescence levels > 200X lower in intensity and were barely visible above the microscope noise. Fluorescence images are maximum intensity projections and scale bars are 5 μm

Fixed sperm were mounted on standard microscope slides using Vectashield hardset mounting media (Vector Laboratories) and #1.5 thickness cover glass. Microscopic imaging was performed using various systems, selected based on the specific requirements for qualitative or quantitative analyses, as well as system availability. Quantitative imaging was performed using an OMX v4 Blaze microscope (GE Healthcare) equipped with a 60X, 1.42 NA oil immersion objective lens and three sCMOS cameras. Initial imaging was performed using structured illumination mode for the best spatial resolution, which employed 568 nm laser excitation (Fig. 2A, C). Brightfield images were also acquired for comparison (Fig. 2B, D. Quantitative imaging was conducted in widefield mode using LED illumination and the TRITC channel. Exposure settings were optimized to ensure sufficient signal compared to the background and coverslip debris, and to prevent camera saturation: for the L-MO group, 30 ms exposures at 100% maximum intensity were used, while for the F-MO group, 50 ms exposures at 31.3% maximum intensity were applied due to the overall higher fluorescence intensity. To ensure accurate comparisons, intensity values were proportionally adjusted relative to the total light dose during the analysis. All acquired images were deconvolved using the proprietary software provided by the microscope manufacturer, and representative images were selected for inclusion in the figures. Post fertilization, salmon embryos were imaged using Zeiss SteREO Lumar.V12 microscope equipped with an ApoLumar S 1.2x objective lens and a red filter set (43CY3, Exitation/Emission 545/605 nm).

### Quantitative analysis of fluorescence intensity in spermatozoa

Fluorescence intensity of spermatozoa incubated with unencapsulated MO (F-MO) or liposome-encapsulated MO (L-MO) was analyzed using Fiji/ImageJ software (ImageJ 1.50i, March 2016) [28], while representative images for the manuscript were prepared using version 1.54p. Quantitative analysis was performed on raw images, while deconvolved and reconstructed structured illumination images were used for figures. A total of 100 sperm cells from the L-MO group and 114 from the F-MO group were analyzed.

Fluorescence intensities were measured in five regions of each sperm cell at the central plane: background intensity (BI), head membrane (HM), tail region (TO), inside the head (IH), and connecting piece (at the base of the sperm head) region (TI). For HM and TO, line profiles (*n* = 5) were drawn orthogonally across the fluorescent regions, and maximum intensities were recorded. For TI and IH, intensities were measured within defined areas, with maximum or mean values used for analysis. Background intensity was measured from the same planes, and corrected fluorescence levels were calculated by subtracting BI from the measured intensities and expressed as normalized fold intensity and mean intensity ratios for the different regions of individual sperm cells. Normalization for different acquisition settings was performed after background subtraction. ImageJ macros which were used to support measuring IH and background values are available in the supplemental material. These values were used to assess the distribution profile and uptake preferences of F-MO and L-MO between treatment groups.

### In vitro fertilization

The fertilization potential of pre-treated spermatozoa was evaluated in the laboratory using in vitro fertilization experiments with an aim of sperm-mediated molecular delivery to eggs.

Eggs (100–120 per replicate) were used for fertilization with surplus pre-treated spermatozoa using sperm: egg ratio of 7.4 × 10^5^. Fertilization experiments were conducted in triplicate following the protocol described by Kumari et al. (2017) [23]. Briefly, 100 µL of treated sperm (~ 7.4 × 10^7^ spermatozoa) was added to the eggs in a 250 mL plastic beaker. The eggs and sperm were gently mixed by adding 25 mL of dechlorinated tap water, followed by a two-minute incubation. Subsequently, 200 mL of dechlorinated tap water was carefully added to the beaker without disturbing the eggs, and the mixture was left undisturbed at 7 °C for a minimum of 4 h to allow for complete ‘swelling’ (water uptake into the perivitelline space).

To assess the success of sperm-mediated molecular delivery of unencapsulated MO (F-MO) and liposome-encapsulated MO (L-MO), 10 eggs were sampled at the one-cell stage, 6 h post-fertilization (~ 2 day degree-days, dd- temperature x number of days). The embryos were examined under a Zeiss SteREO Lumar.V12 fluorescence microscope as mentioned in the previous section. All imaging parameters, including settings and exposure times, were kept consistent across all treatments and controls. Following fertilization, the remaining eggs were transferred to an 8-tray egg incubator with running water maintained at 7 °C in darkness until hatching. Egg mortality was recorded at regular intervals throughout development until the “eyed stage,” corresponding to approximately 300 dd of development [29], to evaluate fertilization efficiency and the developmental capacity of pre-treated sperm with F-MO or L-MO.

### Imaging figure preparation

**Fig. 3:**
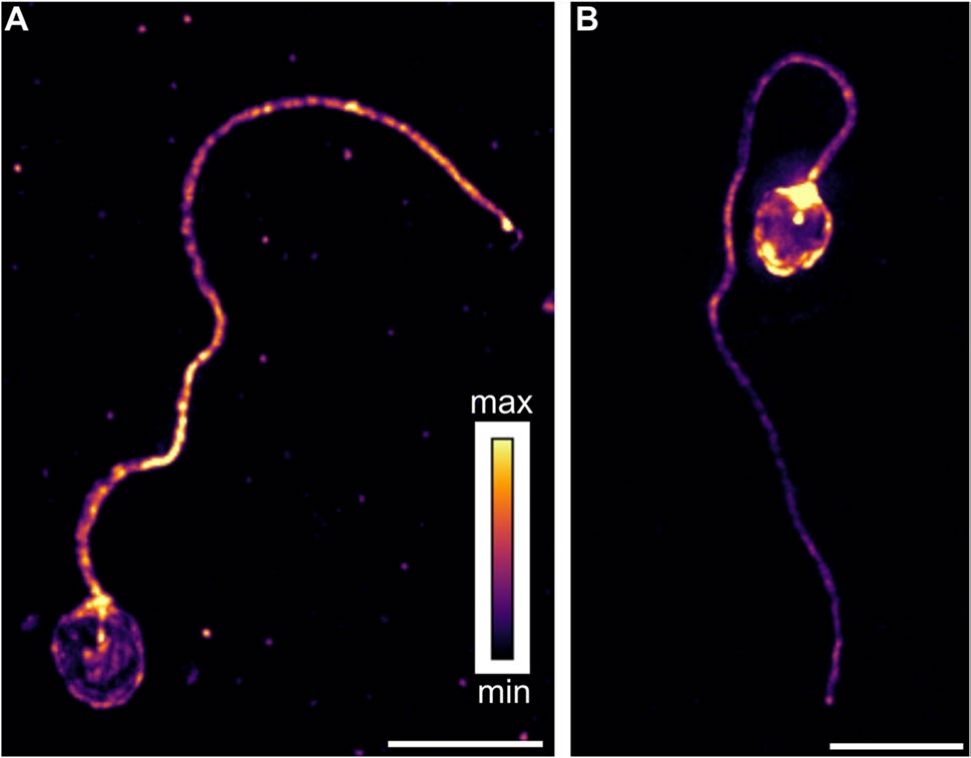
Characteristic localization of cholesterol-conjugated and lissamine-labeled L-MO and F-MO. Sperm treated with L-MO (**A**) showed the highest intensities (in yellow) in the mid-piece region, followed by the tail region, then the head membrane, and with the lowest intensities inside the head (in purple-black). For sperm treated with F-MO (**B**), the head membrane demonstrated higher signal than the tail region. These images are maximum intensity projections of 3D data that are representative of the trends seen within the two cell populations. Scale bars are 5 μm, and the color key shows relative intensities for both images

Figure 2A, C were first reconstructed as standard for structured illumination images and Fig. 3 images were deconvolved, both using the SoftWoRx v7.0.0 provided by the microscope manufacturer, prior to further processing for use in the figures. All microscopy images presented in the figures have been prepared using standard tools in FIJI/ImageJ. Figures 2A and C and 3 are maximum intensity projections of 3D data, with a few lower planes removed from the image before projection as needed to reduce background from dye stuck to the coverslip. Brightness and contrast were adjusted for visibility, and lookup tables were chosen to be color-blind friendly. Videos were generated using Volocity v6.3 (Perkin Elmer).

### Statistical data analysis

The normalized fluorescent intensity data obtained were log-transformed before statistical analysis. Multiple comparisons test were performed with normalized fluorescent intensity data and fertilization efficiency data using one-way analysis of variance (ANOVA) followed by Tukey multiple comparison *post hoc* test using SPSS 25.0 software. Differences were considered statistically significant when *P* < 0.05. Data is presented as mean ± standard error of the mean (SEM), unless specified otherwise. Graph were made using Microsoft Excel.

## Result

### Characterization of liposomes

The different liposomal formulations (L-MO and L-Em) size, size distributions and zeta potentials are shown in Table 1. Both L-MO and L-Em liposomes had an average diameter of 96.6 and 74.6 nm, respectively. Both types of liposomes had a neutral charge, as confirmed by the zeta potential measurements. The encapsulation of F-MO did not change the value of the surface charge. The low polydispersity indexes (P.I.) for the liposome types showed that the liposomes were monodisperse, and the incorporation of F-MO did not alter the physical stability of the liposomal vesicles. Encapsulation efficiency of F-MO within the liposomes was about 76%.

**Table 1:** Liposome characteristics. Mean size distribution with polydispersity index (P.I.) and ζ-potential for liposome types. Encapsulation efficiency of fluorescent MO into liposomes is also shown

Types of liposomes	Mean diameter (nm) ± SD	*P*.I.	Zeta (ζ) potential (mV) ± SD	Encapsulation Efficiency (EE%)
Empty liposomes (L-Em)	74.6 ± 44.6	0.36	−1.77 ± 5.7	-
L-MO	96.6 ± 49.6	0.26	−1.27 ± 3.4	76

The values are the averages of three measurements (mean ± SD)

### Molecular uptake of salmon sperm

High-resolution microscopy revealed that both F-MO and L-MO were successfully bound to or taken up by salmon spermatozoa (Figs. 2 and 3, Supplementary Video 1 and 2). Close to 100% of the sperm cells displayed a fluorescent signal, indicating successful binding and uptake of the fluorescently labeled MOs. Moreover, fluorescent signals were observed throughout the sperm head and the tail region (Fig. 3). On the other hand, control sperm without MO or empty liposomes (L-Em) did not show significant fluorescent signals, compared to MO-treated sperm (Supplementary Fig. S2).

### Regional distribution and uptake patterns/preferences of free and liposome-encapsulated MO

**Fig. 4:**
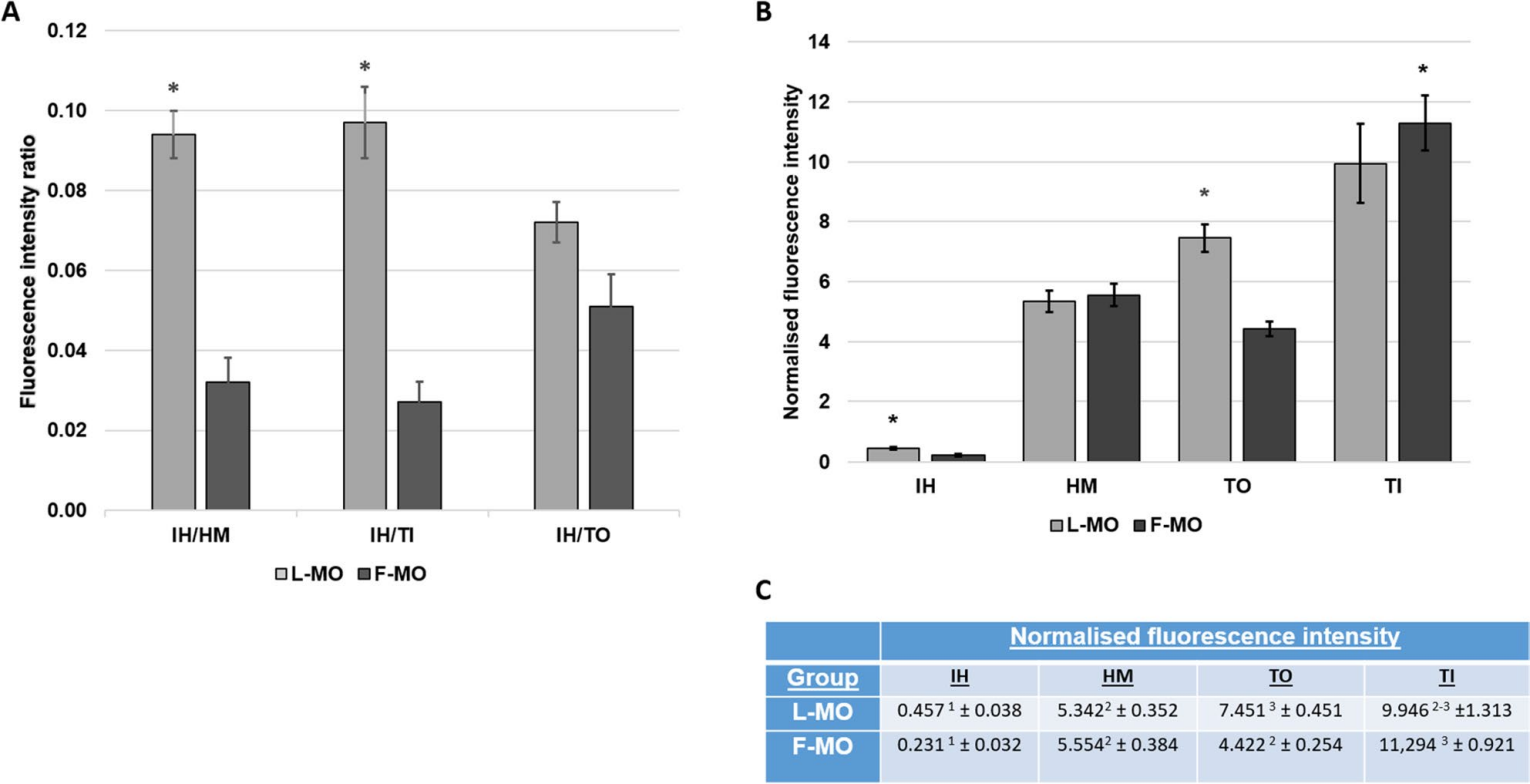
Distribution profile of MO uptake. **A** Fluorescent intensity ratios between different regions of the individual salmon sperm. **B** Normalised fluorescence intensity of the treated sperm population for each subcellular region. Statistical differences (*P* < 0.001) between different L-MO and F-MO are indicated by asterisk (*) above the bars for 4 A and 4B. **C** Table shows normalised fluorescence intensity values in each sub-cellular region as represented in graph B. The superscript in the table with numbers (1, 2, 3) shows significant difference (*P* < 0.0001) in fluorescence intensities between different regions of the sperm (IH, HM, TI, TO) within L-MO or F-MO group. [IH = inside the sperm head, HM = head membrane, TI = connecting piece (at the base of the sperm head), TO = tail region]. All the data shown in 4 **A**, **B**, and **C** are normalised to the background fluorescence intensities using the formula (mean-background/background) and expressed in mean ± SEM (*n* = 100–114)

A total of 100 sperm cells from L-MO and 114 sperm cells from F-MO treated groups were analyzed for fluorescent intensity and the distribution pattern/profile of fluorescent MO attached and internalized within the sperm cell. The result showed distinct differences in the regional distribution of fluorescence between the two delivery methods. L-MOs (Fig. 3A; Supplemental Video 1) exhibited a stronger preference for connecting piece (at the base of the sperm head) region (TI), followed by tail region (TO), head membrane (HM), and then inside the sperm head (IH). In contrast, F-MOs (Fig. 3B; Supplemental Video 2) showed the strongest preference for the connecting piece (TI), followed by HM, TO, and IH as quantitatively shown in Fig. 4B and C. While both F-MO and L-MO groups demonstrated nuclear internalization, the fluorescence intensity in IH was significantly higher in the L-MO group compared to the F-MO group (Fig. 4B and C). Representative images (Fig. 3) and quantitative individual sperm signal intensity ratio (Fig. 4A) further illustrate these differences, highlighting the enhanced uptake and higher internalization inside the head region, IH of L-MO compared to F-MO.

### Sperm mediated delivery of labeled MO during fertilization

**Fig. 5:**
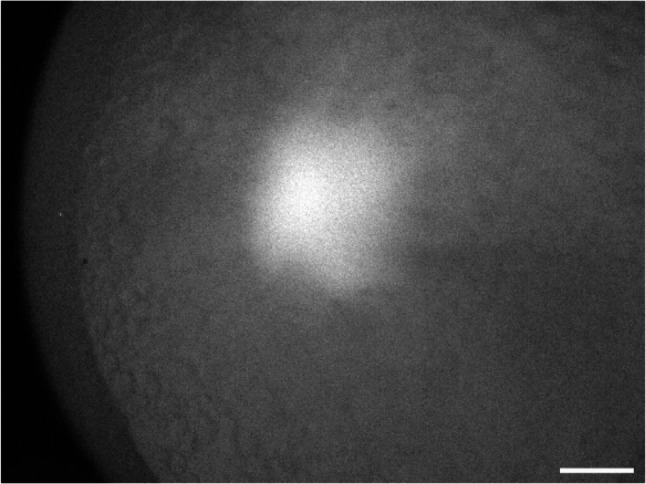
Sperm mediated delivery of L-MO to eggs. A salmon egg is shown 6 h post fertilization with L-MO loaded sperm. Here the yolk granules are visible surrounding the first cell. An untreated control egg was also imaged but showed no fluorescence (corresponding empty image is available as Supplemental Fig. 1). The image was taken using a red (Cy3) filter, and the scale bar is 500 μm

To determine whether MO-loaded sperm could deliver their cargo to the egg during fertilization, embryos were examined at the one-cell stage, 6 h post-fertilization. A positive fluorescence signal was detected in the first cell of embryos fertilized with L-MO-treated sperm but not with F-MOs (Fig. 5). Control group spermatozoa treated under similar conditions with PBS/L-Em did not show any fluorescence (Supplementary Fig. 1).

### Fertilization efficiency and early development

To ensure that the MO treatment did not compromise sperm functionality, fertilization efficiency and the developmental capacity of the pre-treated sperm were evaluated. Under the experimental conditions, no statistically significant differences were found between treatments (L-MO-90.8%, F-MO-86.5%) and untreated sperm (89.1%) in terms of fertilization success or developmental capacity (developmental arrest or deformities) of the embryos up to the “eyed stage” (300 dd) (*P* > 0.05, one-way ANOVA).

## Discussion

This study demonstrates the feasibility of using salmon spermatozoa as a vehicle for the delivery of a liposome-encapsulated MOs (L-MO) to Atlantic salmon eggs. The findings highlight the potential of liposome carriers to enhance the uptake and intracellular distribution of MOs in salmon sperm, offering a promising approach for molecular delivery in aquaculture.

### Molecular uptake and intracellular distribution of MOs

Spermatozoa of higher vertebrates have been shown to fuse and take up encapsulated liposomes [18, 30]. However, the efficiency of molecular uptake and internalization is highly species-specific, influenced by factors such as sperm membrane composition, lipid content, and incubation conditions [31, 32]. Reduced fluorescent binding using shorter (two hours), but no improvement using longer (48 h) incubation time during preliminary trials, indicate that salmon spermatozoa become “saturated” or reach an equilibrium with the surrounding MO variants sometime between 2 and 24 h of incubation at 4 °C at a specific MO concentration. This is substantially longer compared to the incubation times (60–90 min) at higher temperatures reported in mammals and other aquatic species [9, 12, 32]. This highlights the importance of optimizing incubation conditions, including temperature, concentration and duration, for each species to achieve efficient molecular delivery [18].

Our results showed that both free cholesterol-conjugated MOs (F-MO) and the same MO encapsulated into liposomes (L-MO) were successfully fused or internalized into salmon spermatozoa, with nearly 100% sperm cells displaying fluorescence signals (Fig. 2). However, the regional distribution of fluorescence differed significantly between the two delivery methods. L-MO exhibited a stronger preference for the connecting piece region at the base of the sperm head region (TI) and the internal region of the sperm head (IH), indicating more efficient internalization of L-MOs to the sperm nucleus. In contrast, F-MO showed relatively higher fluorescence in the tail (TO) and the head membrane (HM) regions, suggesting that free MOs primarily attach to the sperm membrane with limited nuclear internalization (Figs. 3 and 4). Overall, these findings suggest that small unilamellar liposome encapsulation enhances the ability of MOs to penetrate the sperm cell membrane and accumulates in the sperm head, where the nucleus is located, potentially facilitating more efficient cargo transport to the egg during fertilization. Similar studies in boar sperm have shown efficient uptake of fluorescently labelled liposomes [18]. However, the observed fluorescence signals in our study for L-MO could result from either liposome adhesion or fusion with the sperm membrane. Further experiments such as dequenching assays are needed to confirm the exact mechanism of liposome-sperm interaction.

The observed differences in uptake and distribution patterns may be attributed to the physicochemical properties of the liposomes, including their nanoscale size (~ 97 nm), unilamellar structure and neutral charge. Other factors such as the lipid composition of the sperm membrane and the presence of lipid rafts may play a more significant role in facilitating uptake. Lipid rafts, which are specialized microdomains enriched in cholesterol and sphingolipids, have been suggested as key mediators of exosome uptake in mammalian sperm [33, 34] and may similarly facilitate the interaction and internalization of liposomes in salmon sperm. Additionally, the size of the liposomes (~ 97 nm) is considerably larger than the unencapsulated F-MO conjugate (~ 9 kDa), which may influence their interaction with the sperm membrane. The exact mechanism of uptake remains unclear, but it is plausible that liposome encapsulation enhances the stability and bioavailability of the cargo, allowing for more efficient internalization and intracellular distribution. Further studies are needed to elucidate the specific mechanisms underlying liposome-sperm interactions, including the potential role of lipid rafts. Similar findings have been reported in studies on higher vertebrates, where liposome-mediated delivery systems improved the uptake of exogenous molecules without compromising sperm functionality [14, 35, 36]. These results underscore the importance of liposome encapsulation as a strategy to enhance the efficiency of molecular delivery in sperm-mediated systems, particularly for applications in aquaculture.

### Sperm fertilization potential

An important consideration for any sperm-mediated molecular delivery system is its impact on sperm functionality and fertilization potential. In this study, there are no noticeable differences in fertilization efficiency or embryonic development between treated and untreated sperm when using a surplus sperm-to-egg ratio, comparable to the standard ratio employed in the salmon industry for routine production. These findings suggest that the molecular treatments, including liposome encapsulation, did not adversely affect the ability of sperm to fertilize eggs or support normal embryonic development under the conditions tested. Our results are consistent with previous research on sperm-mediated molecular transfer. For instance, Bachiller et al. [35] demonstrated that liposome-treated sperm could efficiently deliver DNA without impairing fertilization in mice. Similarly, studies on human spermatozoa have shown that liposome attachment or the introduction of exogenous DNA does not compromise physiological or motility attributes and can enhance molecular uptake without affecting gamete activity [37, 38]. While these findings provide valuable insights, it is important to note that most of these studies have been conducted in mammals, which display a distinct difference in sperm biology and fertilization mechanisms compared to those of fish. In fish, external fertilization and the unique composition of sperm membranes may influence the uptake and delivery of molecular cargo. Therefore, further studies are required to comprehensively evaluate sperm functionality in fish species, particularly focusing on motility patterns and sperm physiology post molecular treatments. Such studies will be critical to optimizing sperm-mediated molecular delivery systems for aquaculture applications and ensuring their compatibility with large-scale production practices.

### Sperm-mediated delivery during fertilization

The detection of fluorescence signals in the one-cell stage of embryos fertilized with L-MO-treated sperm (Fig. 5) confirms the successful delivery of liposome-encapsulated non-specific control MOs to the single cell embryo. However, due to logistical reasons, including the need to transport embryos to a separate facility for imaging, it was not possible to track the fluorescent distribution during later developmental stages which was particularly sensitive to stress. Conversely, no fluorescence signals were observed in the one-cell stage embryos fertilized with F-MO treated sperm, indicating differences in the efficiency of molecular delivery between F-MO and L-MO loaded spermatozoa. Nevertheless, we cannot entirely rule out the possibility that F-MO was internalized at levels below the detection threshold of fluorescence imaging.

While this study establishes proof of concept for the successful delivery of liposome-encapsulated non-specific control MOs, as evidenced by positive fluorescent signals in the one-cell stage embryos, it does not provide direct evidence of MO delivery into the oocyte in terms of translational changes, biological function, or a resulting specific phenotype/morphant. This limitation arises from the use of a non-specific control MO, which was not designed to target any specific gene. Consequently, no changes in gene expression or protein translation were anticipated in this study.

## Conclusion

The key finding of this study is that the intracellular distribution of antisense molecules in sperm, whether bound to the membrane or internalized, plays a critical role in determining the efficiency of molecular transfer to the egg. The significantly higher internalization of liposome-encapsulated MOs (L-MO) compared to unencapsulated MOs (F-MO) underscores the importance of using delivery systems such as liposomes or nanoparticles to enhance the transport of antisense molecules. This is particularly relevant for larger molecules, such as protein-coding mRNAs, which may require efficient encapsulation and internalization for successful delivery to the egg. These findings highlight the potential of liposome-mediated delivery systems for improving the efficiency of sperm-mediated molecular transfer. While this study demonstrates the successful delivery of liposome encapsulated cholesterol conjugated morpholino oligonucleotides (L-MO) to the egg via sperm-mediated transfer, it does not establish whether the amount of MO delivered to the zygote is sufficient to achieve biologically relevant antisense activity. This limitation is associated with the proof of concept nature of the study, as control MOs used as a model do not target any specific gene. Future studies using gene-specific MOs with optimized concentration, capable of modulating targeted gene expression will be necessary to determine whether the delivered MO levels reach an effective threshold for functional activity in the embryo. Such advancements could pave the way for the development of more effective and scalable molecular delivery systems in aquaculture and other fields of reproductive biology.

## Supplementary Information

Supplementary Material 1.

Supplementary Material 2.

Supplementary Material 3.

## Data Availability

The datasets used and/or analyzed during the current study are available from the corresponding author on reasonable request.

## Abbreviations

ASO: Antisense oligonucleotides
MO: Morpholino
dd: Day degrees
ANOVA: One-way analysis of variance
SEM: Standard error of the mean

## References

[1] Ekker SC, Larson JD. Morphant technology in model developmental systems. Genesis. 2001;30:89–93. 11477681 10.1002/gene.1038

[2] Szukowska A, Żuk M, Sztompke J, Bednarz B, Kaźmierczak U. Application of antisense oligonucleotides as an alternative approach for gene expression control and functional studies. Int J Mol Sci. 2025;26(21):10524. 41226561 10.3390/ijms262110524 PMC12610768

[3] Xu L, Zhao M, Ryu JH, Hayman ES, Fairgrieve WT, Zohar Y, Luckenbach JA, Wong TT. Reproductive sterility in aquaculture: A review of induction methods and an emerging approach with application to Pacific Northwest finfish species. Reviews Aquaculture. 2023;15(1):220–41. . 10.1111/raq.12712

[4] Lanes CF, Marins LF. Sperm-mediated gene transfer in aquatic species: Present, past and future. In Sperm-Mediated Gene Transfer: Concepts and Controversies 2012 May 25 (pp. 64–75). Bentham Science.

[5] Collares T, Campos VF, Seixas FK, Cavalcanti PV, Dellagostin OA, Moreira HL, Deschamps JC. Transgene transmission in South American catfish (*Rhamdia quelen*) larvae by sperm-mediated gene transfer. J Biosci. 2010;35(1):39–47. 20413908 10.1007/s12038-010-0006-6

[6] Coward K, Kubota H, Parrington J. vivo gene transfer into testis and sperm: developments and future application. Arch Androl. 2007;53(4):187–97. 17852043 10.1080/01485010701426455

[7] Barchanski A, Taylor U, Sajti CL, Gamrad L, Kues WA, Rath D, Barcikowski S. Bioconjugated gold nanoparticles penetrate into spermatozoa depending on plasma membrane status. J Biomed Nanotechnol. 2015;11(9):1597–607. 26485929 10.1166/jbn.2015.2094

[8] Sciamanna I, Vitullo P, Curatolo A, Spadafora C. Retrotransposons, reverse transcriptase and the genesis of new genetic information. Gene. 2009;448(2):180–6. 19631262 10.1016/j.gene.2009.07.011

[9] Brackett BG, Baranska W, Sawicki W, Koprowski H. Uptake of heterologous genome by mammalian spermatozoa and its transfer to ova through fertilization. Proceedings of the National Academy of Sciences. 1971;68(2):353-7. 10.1073/pnas.68.2.353 PMC388936 5277085

[10] Khoo HW. Sperm-mediated gene transfer studies on zebrafish in Singapore. Mol Reprod Development: Incorporating Gamete Res. 2000;56(S2):278–80. 10.1002/(SICI)1098-2795(200006)56:2+<278::AID-MRD14>3.0.CO;2-A 10824984

[11] Lu JK, Fu BH, Wu JL, Chen TT. Production of transgenic silver sea bream (*Sparus sarba*) by different gene transfer methods. Mar Biotechnol. 2002;4(3):328–37. 10.1007/s10126-002-0027-8 14961266

[12] Lavitrano M, Camaioni A, Fazio VM, Dolci S, Farace MG, Spadafora C. Sperm cells as vectors for introducing foreign DNA into eggs: genetic transformation of mice. Cell. 1989;57(5):717–23. 2720785 10.1016/0092-8674(89)90787-3

[13] Jonak J. Sperm-mediated preparation of transgenic Xenopus laevis and transmission of transgenic DNA to the next generation. Mol Reprod Development: Incorporating Gamete Res. 2000;56(S2):298–300. 10.1002/(SICI)1098-2795(200006)56:2+<298::AID-MRD19>3.0.CO;2-Q 10824989

[14] Parrington J, Coward K, Gadea J. Sperm and testis mediated DNA transfer as a means of gene therapy. Syst biology reproductive Med. 2011;57(1–2):35–42. 10.3109/19396368.2010.514022 21222517

[15] Gupta R, Salave S, Rana D, Karunakaran B, Butreddy A, Benival D, Kommineni N. Versatility of liposomes for antisense oligonucleotide delivery: a special focus on various therapeutic areas. Pharmaceutics. 2023;15(5):1435. 37242677 10.3390/pharmaceutics15051435 PMC10222274

[16] Nsairat H, Khater D, Sayed U, Odeh F, Al Bawab A, Alshaer W. Liposomes: structure, composition, types, and clinical applications. Heliyon. 2022;8(5). 10.1016/j.heliyon.2022.e09394 PMC9118483 35600452

[17] Davidson LM, Barkalina N, Coward K. Development of nanoparticle-mediated delivery tools to investigate the role of molecular genetic mechanisms underlying male infertility. Sci Adv Today. 2016;1:25210.

[18] Kasimanickam VR, Buhr MM. Fusion of boar sperm with nanoliposomes prepared from synthetic phospholipids. Reprod Domest Anim. 2016;51(4):461–6. 27217373 10.1111/rda.12702

[19] Gregoriadis G. The liposome drug-carrier concept: its development and future. Liposomes Biol Syst. 1980:25–86.

[20] Vaage J, Donovan D, Loftus T, Uster P, Working P. Prophylaxis and therapy of mouse mammary carcinomas with doxorubicin and vincristine encapsulated in sterically stabilised liposomes. Eur J Cancer. 1995;31(3):367–72. 10.1016/0959-8049(94)00443-9 7786604

[21] Hara T, Aramaki Y, Takada S, Koike K, Tsuchiya S. Receptor-mediated transfer of pSV2CAT DNA to a human hepatoblastoma cell line HepG2 using asialofetuin-labeled cationic liposomes. Gene. 1995;159(2):167–74. 7542617 10.1016/0378-1119(95)00100-k

[22] Garrett FE, Goel S, Yasul J, Koch RA. Liposomes fuse with sperm cells and induce activation by delivery of impermeant agents. Biochim et Biophys Acta (BBA)-Biomembranes. 1999;1417(1):77–88. 10.1016/s0005-2736(98)00258-2 10076037

[23] Kumari J, Flaten GE, Škalko-Basnet N, Tveiten H. Molecular transfer to Atlantic salmon ovulated eggs using liposomes. Aquaculture. 2017;479:404–11.

[24] Li YF. Functionalizing Morpholino oligos for antisense drug research and development. J Drug Discov Develop Deliv. 2016;3(1):1021.

[25] Østergaard ME, Jackson M, Low A, Chappell E, Lee AG, Peralta R, Yu RQ, Kinberger J, Dan GA, Carty A, Tanowitz R. Conjugation of hydrophobic moieties enhances potency of antisense oligonucleotides in the muscle of rodents and non-human primates. Nucleic Acids Res. 2019;47(12):6045–58. 31076766 10.1093/nar/gkz360 PMC6614849

[26] Naderkhani E, Erber A, Škalko-Basnet N, Flaten GE. Improved permeability of acyclovir: optimization of mucoadhesive liposomes using the phospholipid vesicle-based permeation assay. J Pharm Sci. 2014;103(2):661–8. 24395733 10.1002/jps.23845

[27] Jøraholmen MW, Škalko-Basnet N, Acharya G, Basnet P. Resveratrol-loaded liposomes for topical treatment of the vaginal inflammation and infections. Eur J Pharm Sci. 2015;79:112–21. 26360840 10.1016/j.ejps.2015.09.007

[28] Schindelin J, Arganda-Carreras I, Frise E, Kaynig V, Longair M, Pietzsch T, Preibisch S, Rueden C, Saalfeld S, Schmid B, Tinevez JY. Fiji: an open-source platform for biological-image analysis. Nat Methods. 2012;9(7):676–82. 22743772 10.1038/nmeth.2019 PMC3855844

[29] Musialak LA, Finstad B, Bråthen KE, Kjørsvik E. Embryonic development and sensitive stages of Atlantic salmon (Salmo salar) eggs. Aquaculture. 2024;579:740281.

[30] Arts EG, Kuiken J, Jager S, Hoekstra D. Fusion of artificial membranes with mammalian spermatozoa: specific involvement of the equatorial segment after acrosome reaction. Eur J Biochem. 1993;217(3):1001–9. 8223623 10.1111/j.1432-1033.1993.tb18331.x

[31] Anzar M, Kakuda N, He L, Pauls KP, Buhr MM. Optimizing and quantifying fusion of liposomes to mammalian sperm using resonance energy transfer and flow cytometric methods. Cytometry: J Int Soc Anal Cytol. 2002;49(1):22–7. 10.1002/cyto.10137 12210607

[32] Drokin SI. Phospholipids and fatty acids of phospholipids of sperm from several freshwater and marine species of fish. Comp Biochem Physiol Part B: Comp Biochem. 1993;104(2):423–8.

[33] Suhaiman L, Belmonte SA. Lipid remodeling in acrosome exocytosis: unraveling key players in the human sperm. Front Cell Dev Biology. 2024;12:1457638. 10.3389/fcell.2024.1457638 PMC11456524 39376630

[34] Hickey KD, Buhr MM. Lipid bilayer composition affects transmembrane protein orientation and function. J lipids. 2011;2011(1):208457. 21490797 10.1155/2011/208457 PMC3068514

[35] Bachiller D, Schellander K, Peli J, Rüther U. Liposome-mediated DNA uptake by sperm cells. Mol Reprod Dev. 1991;30(3):194–200. 1793596 10.1002/mrd.1080300305

[36] Barkalina N, Jones C, Townley H, Coward K. Functionalization of mesoporous silica nanoparticles with a cell-penetrating peptide to target mammalian sperm in vitro. Nanomedicine. 2015;10(10):1539–53. 26008192 10.2217/nnm.14.235

[37] Geerts N, McGrath J, Stronk JN, Vanderlick TK, Huszar G. Spermatozoa as a transport system of large unilamellar lipid vesicles into the oocyte. Reprod Biomed Online. 2014;28(4):451–61. 24581981 10.1016/j.rbmo.2013.11.009

[38] Barkalina N, Jones C, Wood MJ, Coward K. Extracellular vesicle-mediated delivery of molecular compounds into gametes and embryos: learning from nature. Hum Reprod Update. 2015;21(5):627–39. 26071427 10.1093/humupd/dmv027

