# Supplementary material for: Delivery and uptake of free and liposome-encapsulated cholesterol-conjugated antisense oligonucleotides in Atlantic salmon sperm: insights from high-resolution imaging
Source: BMC Vet Res. 2026 Apr 17;22:318. doi: 10.1186/s12917-026-05465-0 (PMC13217789; doi:10.1186/s12917-026-05465-0)
Supplement: Supplementary file 3 — Supplementary Material 3. [file 12917_2026_5465_MOESM3_ESM.docx]

**Supplementary Figure 1**

Salmon egg, 6 h post fertilization with sperm loaded with empty liposome (control group) correlating to Figure 5, using the same red imaging channel. Here, fluorescence is so low that effectively only noise is visible in the image. Scale bar is 500 µm.


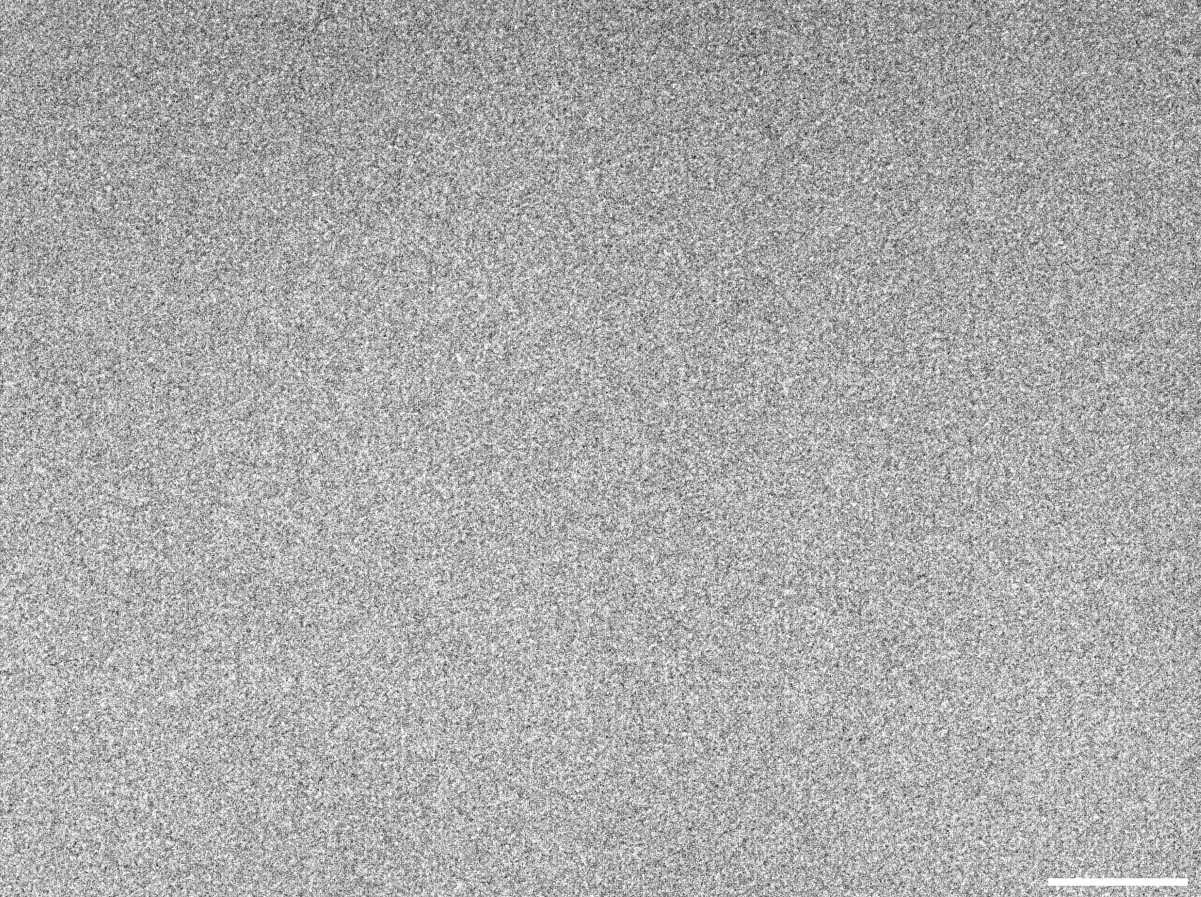


**Supplementary Figure 2**

Fluorescence intensity of control sperm. The control sperm image (B) shows no detectable fluorescence signal when using the same contrast adjustments as the MO incubated sperm image (A), indicating that the relative fluorescence intensity of the control sperm is effectively negligible. All images are maximum intensity projections of widefield fluorescence microscopy captured under identical microscope settings. Scale bar is 10 µm.

**
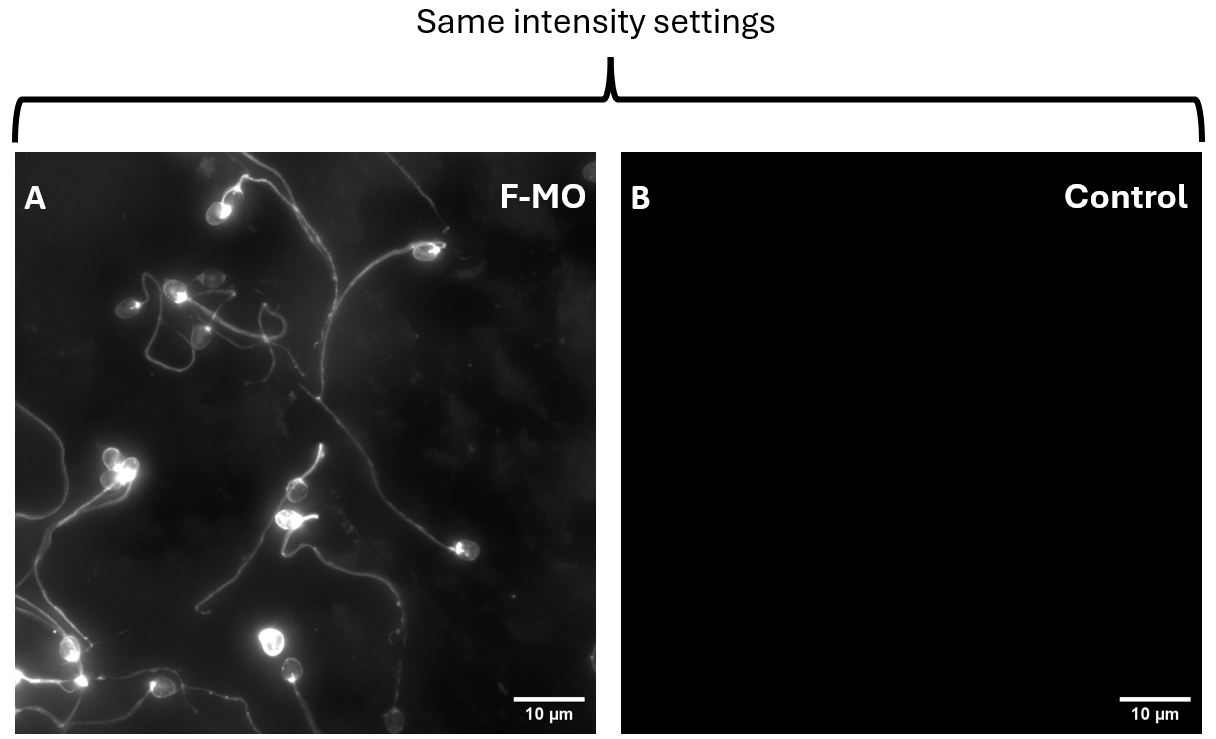
**

**Supplementary Video 1**

3D rendering of L-MO treated sperm, imaged using fluorescence deconvolution microscopy. The video starts with the full field of view, then zooms in and rotates around the sperm head.

**Supplementary Video 2**

3D rendering of F-MO treated sperm, imaged using fluorescence deconvolution microscopy. The video starts with the full field of view, then zooms in and rotates around the sperm head.

**ImageJ Macros used for quantitative analysis**

Additional image analysis was performed manually as described in the methodology section.

macro "SalmSperm Inner [i]" {

title = getTitle()

getPixelSize(pixelunits, pix1, pix2);

setTool("line")

waitForUser("Choose the middle plane. Draw a line from the end of the invagination to the end of the cell")

getLine(x1,y1,x2,y2,w)

circlesize = getNumber("Radius of circle to analyze in um", 0.5)

csp=circlesize/pix1

midx=(x1+x2)/2;

midy=(y1+y2)/2;

startx=midx-csp;

starty=midy-csp;

//print (x1,x2,startx,y1,y2,starty)

makeOval(startx, starty, (csp)*2, (csp)*2);

run("Measure");

}

macro "SalmSperm Background [b]" {

title = getTitle()

getPixelSize(pixelunits, pix1, pix2);

circlesize = getNumber("Radius of circle to analyze in um", 0.5)

csp=circlesize/pix1

setTool("rectangle");

waitForUser("Choose the middle plane. Pick a rectangle whose corners include your background points");

getSelectionBounds(x, y, w, h)

makeOval(x, y, (csp)*2, (csp)*2);

run("Measure");

makeOval(x+w, y, (csp)*2, (csp)*2);

run("Measure");

makeOval(x, y+h, (csp)*2, (csp)*2);

run("Measure");

makeOval(x+w, y+h, (csp)*2, (csp)*2);

run("Measure");

centerx=(w/2)+x;

centery=(h/2)+y;

makeOval(centerx, centery, (csp)*2, (csp)*2);

run("Measure");

}

}
